# Association of root morphology of mandibular second molars on panoramic-like and axial views of cone-beam computed tomography

**DOI:** 10.1186/s12903-023-03526-6

**Published:** 2023-11-13

**Authors:** Mahsa Karkhaneh, Hamed Karkehabadi, Behnaz Alafchi, Abbas Shokri

**Affiliations:** 1 Department of Pediatric Dentistry, Tabriz University of Medical Sciences, Tabriz, Iran; 2 Department of Endodontics, Dental School, Hamadan University of Medical Sciences, Hamadan, Iran; 3 Department of Biostatistics, School of Public Health and Research Center for Health Sciences, Hamadan University of Medical Sciences, Hamadan, Iran; 4 Dental Implants Research Center, Department of Oral and Maxillofacial Radiology, Dental School, Hamadan University of Medical Sciences, Hamadan, Iran

**Keywords:** Mandibular molar, Cone-Beam Computed Tomography, Panoramic, Root morphology, C-Shaped canal

## Abstract

**Background:**

Knowledge about the anatomy and morphology of the root canal system is essential for successful surgical and non-surgical root canal treatments. However, precise assessment of the root morphology and anatomy is not often possible on two-dimensional radiographs. This study aimed to investigate the association of root morphology of mandibular second molars on panoramic-like and axial views of cone-beam computed tomography (CBCT).

**Methods:**

This cross-sectional study evaluated 1,231 CBCT scans of mandibular second molars obtained between October 2018 and February 2022 that were retrieved from the archives of a private radiology clinic. Panoramic-like images were reconstructed from the CBCT scans. The root morphology of mandibular second molars was classified on panoramic-like images as type 1, 2, 3, 4, or 5. The root pattern on axial CBCT images was classified into three types of single, double and C-shaped. The association of root morphology on panoramic-like and axial CBCT views was analyzed by the Chi-square test and Fisher’s exact test at 0.05 level of significance.

**Results:**

Of all, 62.7% of mandibular second molars were type 1; out of which, 97.3% had a double-root pattern on axial CBCT images. Also, 28.6% of them were type 2; of which, 92.6% had a double-root pattern. Moreover, 3.9% were type 3; of which, 47.9% had a C-shaped pattern; 0.9% were type 4, and 45.5% of them showed a single-root pattern; 3.8% were type 5 with 76.6% of them showing a single-root pattern. The prevalence of C-shaped canals was higher in females, and most C-shaped canals had a C3 pattern.

**Conclusion:**

Root morphology on panoramic-like CBCT views had a strong association with the root canal pattern on axial CBCT views. According to the results, mandibular second molars with a type 3 morphology on panoramic-like CBCT images are highly probable to have a C-shaped canal.

## Background

The root canal system has a wide range of anatomical variations in terms of the number of canals at different levels from the apex, the shape of the root canal, and presence of isthmi or web-like patterns [1]. The C-shaped canal morphology is an anatomical variation caused by a failure in development of the Hertwig’s epithelial root sheath [2]. C-shaped canals have isthmi connecting individual canals, resulting in formation of a shape similar to the letter C [2, 3]. C-shaped canals were first identified by Cox and Cooke [4]. Also, Fan et al. [1] classified the cross-sectional shape of C-shaped canals based on the anatomical continuity of the C shape in five categories (C1, C2, C3, C4 and C5). It has been postulated that genetic and ethnic factors are responsible for the formation of C-shaped canals. Moreover, adaptation of teeth to smaller arches may serve as a contributing factor to fusion of the root and development of C-shaped morphology [2]. C-shaped canals are more common in mandibular second molars [5]. The root canal system of mandibular second molars may have several anatomical variations as follows:

- It may have fused roots with buccal or lingual grooves or with one single canal.
- It may have two separate roots.
- It may have a C-shaped pattern (with a deep buccal or lingual groove) [6].

C-shaped canals have a complex and unpredictable anatomy. The canal shape at the orifice level may be different from that at the mid-root or the apical third. Such variations create challenges for the clinicians during endodontic treatment [4].

The incidence of C-shaped canals in Iranian population is 6.96% .[7].

Its main anatomical characteristic is the presence of fins or isthmuses connecting the individual root canals, while the orifice may appear as a single ribbon-shaped opening with an arc of 180 or more, which creates the canal cross-sectional and 3D shape variable along the root; this morphological complexity creates major challenges with respect to debridement, disinfection and canal filling procedures, which ultimately may influence the prognosis of the root canal treatment [8]. Knowledge about the anatomy and morphology of the root canal system is essential for successful surgical and non-surgical root canal treatments. However, it would be difficult to precisely assess the root canal anatomy on two-dimensional radiographic images such as panoramic and periapical radiographs. This is particularly important in mandibular second molars that have a high prevalence of C-shaped canals. In such complex cases, cone-beam computed tomography (CBCT) may be used as the gold standard to examine the morphology of the root canals. CBCT provides three-dimensional images without the shortcomings of two-dimensional modalities such as distortion and superimposition of structures. Also, CBCT has high diagnostic accuracy comparable to that of conventional computed tomography but with the advantage of lower radiation dose [9–11]. Since CBCT has higher patient radiation dose than the conventional radiography, it cannot be requested for all patients unless there is a clear indication for CBCT (such as complex anatomy or proximity to an impacted tooth) [6, 9].

Limited studies are available on the association of conventional radiography and CBCT data [6]. Thus, the purpose of this study was to investigate the association of root morphology of mandibular second molars on panoramic-like CBCT views with the root canal pattern on axial CBCT images.

## Methods

In this cross-sectional study, informed consent was obtained from all participants or their legal guardians to use their radiographic data for research purposes. The study protocol was also reviewed and approved by the Ethics Committee of Hamadan University of Medical Sciences (IR.UMSHA.REC.1400.666). All procedures were carried out in accordance with the relevant guidelines and regulations.

The study population included 1,231 available CBCT scans of patients retrieved from the archives of a private radiology clinic in Hamadan city. The CBCT scans had been taken between September 2018 and February 2022 for purposes not related to this study such as dental implant treatment, orthodontic treatment, and oral and maxillofacial surgical procedures. We have chosen this timeframe because our organized archive had been created since 2018. All images had been obtained with Cranex 3D CBCT scanner (Soredex, Tuusula, Finland) with the exposure settings of 90 kVp, 10 mA, 14.6 s time, 200-µm voxel size and 8 × 8 cm^3^ field of view(fov), and were examined using OnDemand 3D Dental software (Cybermed, Seoul, South Korea). In CBCT images the smallest voxel size and fov are recommended for evaluation of root canal morphology. To reconstruct the panoramic-like images, the slice thickness was selected between 5 and 15 mm (mean thickness of 10 mm) to achieve the best quality according to the buccolingual inclination of the tooth and root. In case of buccal or lingual tilting of the tooth, a thickness of at least 15 mm was selected. The coronal, sagittal, axial, and axial CBCT views were evaluated with a slice interval of 0.5 mm and a slice thickness of 0.5 mm to assess the root morphology. The images were reviewed by a general dentist (with one year experience), a maxillofacial radiologist (with 15 years’ experience), and an endodontist (with 10 years’ experience).Prior to evaluate the images all examiners were trained and calibrated regarding to CBCT software. Complex cases were discussed until a consensus was reached.

### Inclusion criteria

All optimal quality CBCT images visualizing mandibular second molars with fully developed roots and without root canal therapy, periapical lesions, root fracture, calcified canals, post and core restoration, or open apex were included. All participants that had signed informed consent.

### Exclusion criteria

CBCT images visualizing mandibular second molars with one of the following conditions were excluded from the study:

1. Internal or external root resorption.
2. Root anomalies such as hypercementosis, fusion, concrescenes, severe dilacerations, etc. [12, 13].
3. Patients that didn’t have informed consent.

In CBCT images root anomalies, such as hypercementosis, fusion, concrescences, and severe dilacerations obviously have seen and these anomalies disorganize normal anatomy of root. Complex cases were discussed until a consensus was reached.

Mandibular second molars on panoramic-like views were then classified using the modified classification of Fan et al., [6] as follows:

Type 1: Presence of two separate, divergent or parallel roots with clear trabeculae between them.

Type 2: Presence of two separate converging roots with clear trabeculae between them.

Type 3: Presence of attached roots with mesial and distal canals merging near the apex.

Type 4: Presence of attached roots with two separate canals that do not meet at the apex.

**Fig. 1:**
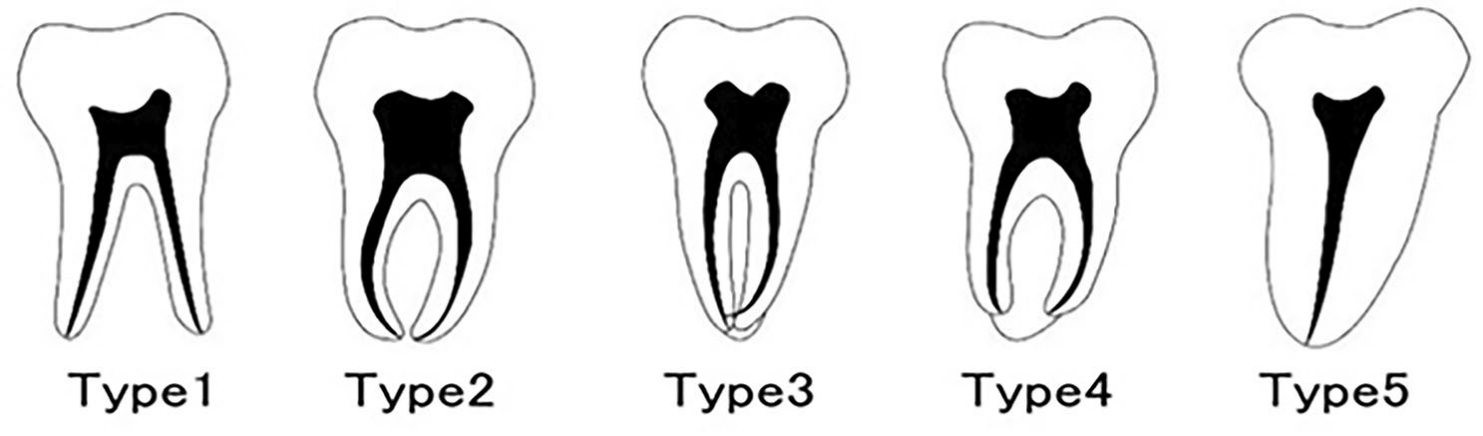
Classification of mandibular second molar tooth morphology on panoramic-like images

Type 5: Presence of one root with one canal (Fig. 1) [6, 13].

On CBCT axial views, the mandibular second molar root canal morphology was classified into three patterns of (I).

single root, (II) double root, and (III) C-shaped.

The single-root pattern refers to presence of a fused root with small grooves on both buccal and lingual surfaces or a round root with one single canal. The double pattern is defined as presence of two separate roots with a trabecular pattern between them. The C-shaped pattern is defined as one root with a deep groove opening only on the buccal or lingual surface compared with the opposite side [6]. Root length was measured on the sagittal sections in three areas of cervical, middle and apical third. The pattern of C-shaped canals was examined on the axial cut and in the cervical, middle and apical sections of the root, and it could have different morphologies at different sections as follows:

C1: It is continuously C-shaped without any branching or separation.

C2: It looks like a semicolon, which is caused by interruption of the C-shaped axis (if it has an angle greater than or equal to 60 degrees).

C3: It is in the form of two or three canals separated from each other along the C-shaped axis (if it has an angle smaller than 60 degrees).

C4: It is seen as a round or oval canal on the axial view.

**Fig. 2:**
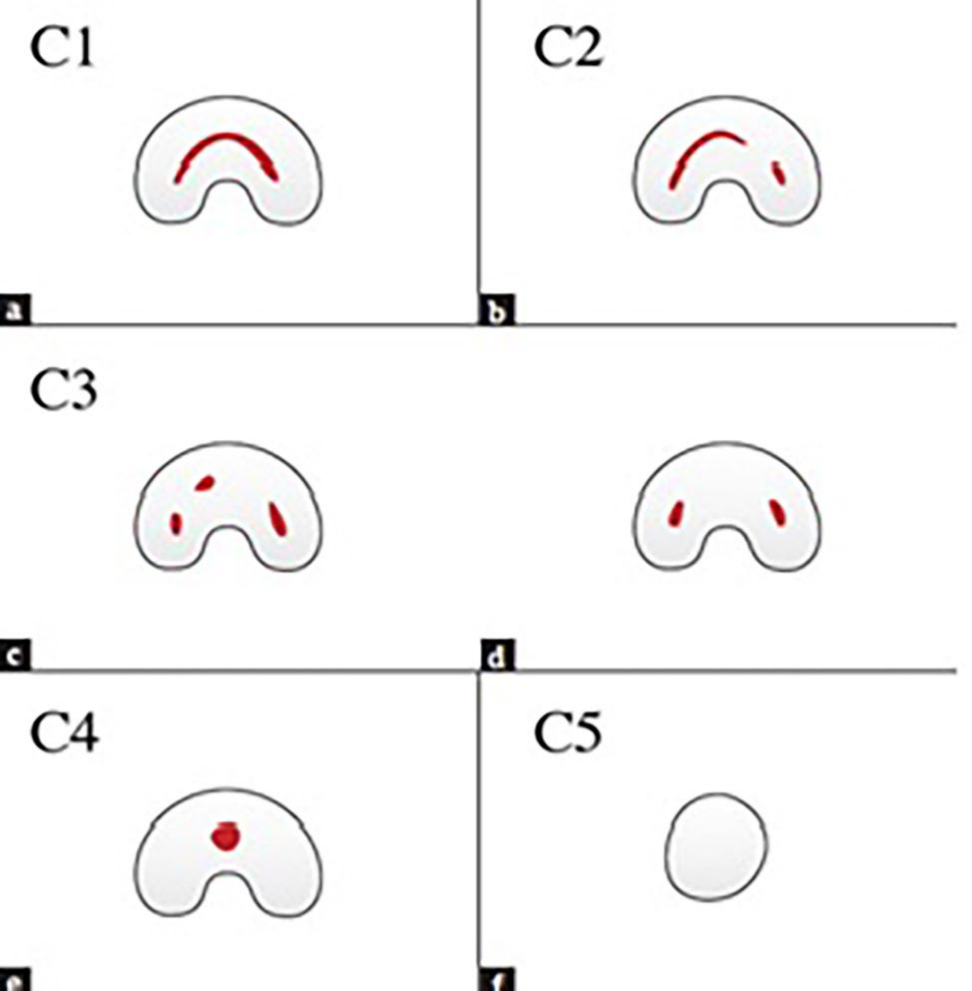
Types of C-shaped canal morphology on the axial CBCT section

**Fig. 3:**
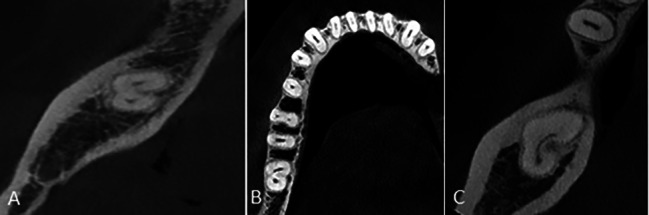
Types of C-shaped canal morphology **(A)** C3, **(B)** C2 and **(C)** C1

C5: No canal is seen, which usually occurs near the apex (Figs. 2 and 3) [1, 14].

The collected data were analyzed by SPSS version 24 (SPSS Inc., IL, USA). The data were presented in frequency tables and graphs, and analyzed by the Chi-square test, and Fisher’s exact test at 0.05 level of significance.

## Results

The intra-observer and interobserver agreements were above 90%, indicating excellent agreement.

In this study, 1231 radiographs including 744 (60.7%) female and 482 (39.3%) male with an average age distribution of 33.87 years were examined.

Demographic information of patients and the association of age and gender with the type of root morphology on panoramic-like views are presented in Tables 1 and 2.

**Table 1:** Association between age and type of root morphology of mandibular second molars on panoramic-like views

Morphology	Mean	Std. deviation	Minimum	Maximum	F	df	P-value
Type 1	34.00	13.34	11.00	80.00			
Type 2	32.97	12.21	13.00	63.00			
Type 3	36.76	14.78	14.00	62.00	1.166	1194	0.324
Type 4	35.60	9.16	23.00	54.00			
Type 5	35.22	10.90	16.00	58.00			
Total	33.87	12.97	11.00	80.00			

**Table 2:** Association between gender and type of root morphology on panoramic-like views

Morphology	Female		Male		Chi-square	df	P-value
	**Number**	**Percentage**	**Number**	**Percentage**			
Type 1	451	60.6%	318	66%			
Type 2	208	28%	143	29.7%			
Type 3	33	4.4%	15	3.1%	23.698	4	**0.0001>**
Type 4	9	1.2%	2	0.4%			
Type 5	43	5.8%	4	0.8%			

The majority of patients with all types of root morphology were women (60.7%).

**Fig. 4:**
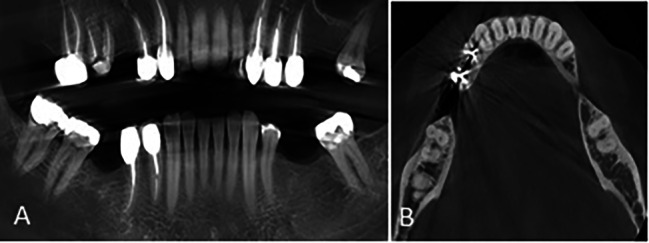
Mandibular second molar tooth with **(A)** type 1 morphology on the panoramic-like image and **(B)** double pattern on the axial CBCT section

The association of root morphology on the panoramic-like view and axial sections was analyzed by the Chi-square test (Table 3). As shown in Table 3, of all 772 cases with type 1 morphology on the panoramic-like view, 751 (97.3%) had a double-root pattern on the axial view (Fig. 4), and this association was statistically significant (P < 0.001).

**Table 3:** Association between the root morphology of mandibular second molars on panoramic-like images and their root canal pattern on axial CBCT sections

Type of morphology on panoramic-like images		Pattern of root in CBCT								Total	Chi-square	df	P-value
		**Single**		**Double**				**C-shaped**					
		**Number**	**Percentage**	**Number**			**Percentage**	**Number**	**Percentage**				
Type 1	1		0.1%		751	97.3%		20	2.6%	772	1421.272	2	> 0.001
Type 2	4		1.1%		327	92.6%		22	6.2%	353	559.994	2	> 0.001
Type 3	21		43.8%		4	8.3%		23	47.9%	48	13.625	2	< 0.001
Type 4	5		45.5%		3	27.3%		3	27.3%	11	0.727	2	0.695
Type 5	36		76.6%		1	2.1%		10	21.3%	47	42.170	2	> 0.001
total	67		5.4%		1086	88.2%		78	6.3%	1231	1669.006	2	> 0.001

**Fig. 5:**
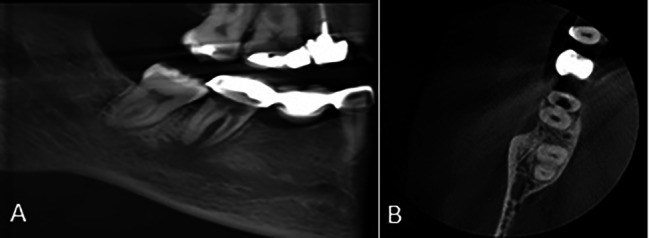
Mandibular second molar tooth with **(A)** type 2 morphology on the panoramic-like image and **(B)** double pattern on the axial CBCT section

Also, of a total of 353 cases with type 2 morphology on the panoramic-like view, 327 (92.6%) had a double-root pattern on the axial view (Fig. 5) and this association was statistically significant (P < 0.001).

**Fig. 6:**
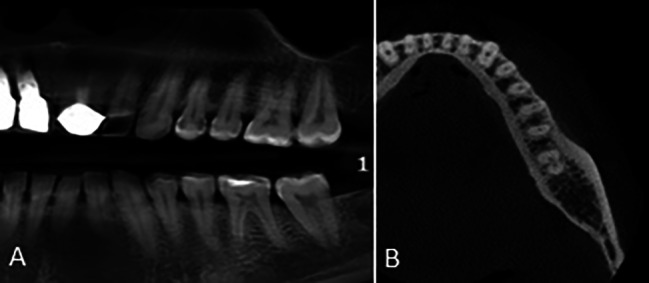
Mandibular second molar tooth with **(A)** type 3 morphology on the panoramic-like image and **(B)** C-shaped pattern (type C3) on the axial CBCT section

Of 48 cases with type 3 morphology on the panoramic-like view, 23 (47.9%) had a C-shaped pattern on the axial view (Fig. 6), and this association was statistically significant (P < 0.001).

**Fig. 7:**
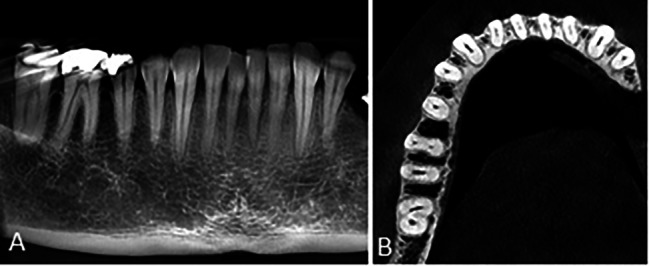
Mandibular second molar tooth with **(A)** type 4 morphology on the panoramic-like image and **(B)** C-shaped (C2) pattern on the axial CBCT section

Of 11 cases with type 4 morphology on the panoramic-like view, 5 (45.5%) had a single-root pattern on the axial view (Fig. 7).

The results showed that although most teeth with type 4 morphology had a single-root pattern, there was no significant association between the type 4 morphology of mandibular second molars on the panoramic-like view and the root canal pattern on the axial view.

**Fig. 8:**
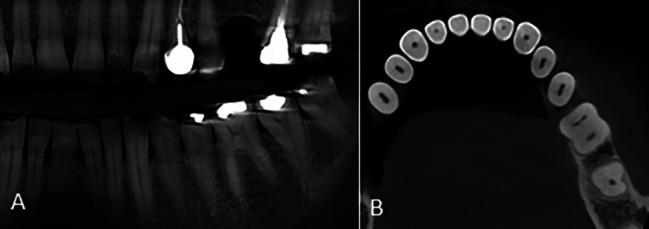
Mandibular second molar tooth with **(A)** type 5 morphology on the panoramic-like image and **(B)** single-root pattern on the axial CBCT section

Of all 47 cases with type 5 morphology on the panoramic-like view, 36 (76.6%) had a single-root pattern on the axial view (Fig. 8).

The results showed that most teeth with type 5 morphology had a single-root pattern on the axial view (P < 0.001).

The association between the type of mandibular second molar root morphology and the type of C-shaped canal on the CBCT scans was analyzed by the Chi-square test and the results are presented in Table 4.

**Table 4:** Association between the root morphology of mandibular second molars on the panoramic-like images and the morphology of C-shaped canals on axial CBCT sections

Type of root morphology																																	
**Type 1**						**Type 2**						**Type 3**							**Type 4**							Type 5							
**Number of C-shaped canals**	**20**						**22**						**23**							**3**							10						
Type of C-shaped canals	C 1	C 2		C 3	C 4	C 5	C 1	C 2	C 3	C 4		C 5	C 1	C 2		C 3	C 4		C 5	C 1	C 2		C 3	C 4		C 5	C 1	C 2		C 3	C 4		C 5
In cervical section	3	0		17	0	0	5	0	16	1		0	11	3		7	2		0	0	0		3	0		0	4	0		2	4		0
In middle section	2	0		18	0	0	4	0	18	0		0	11	3		7	2		0	0	0		3	0		0	1	0		5	4		0
In apical section	2	0		18	0	0	1	0	18	3		0	7	3		9	4		0	0	0		3	0		0	3	0		3	4		0
Chi-square	C		M			A	C		M		A		C		M			A		C		M			A		C		M			A	
	9.800		12.800			12.800	16.455		8.909		23.545		8.826		8.826			3.957									0.800		2.600			0.200	
df	1		1			1	2		1		2		3		3			3									2		2			2	
P-value	**0.002**		**0.001>**			**0.001>**	**0.001>**		**0.003**		**0.001>**		**0.032**		**0.032**			**0.266**									**0.670**		**0.273**			0.905	

df: degree of freedom

C: cervical section

M: middle section

A: apical section

## Discussion

The root canal system may exhibit complex anatomical variations, including C-shaped canals, which have been commonly reported in mandibular second molars. Anatomical irregularities in C-shaped canals, such as lateral and accessory canals or presence of an apical delta, complicate cleaning and shaping, and obtaining a three-dimensional seal [15]. Causes of endodontic failure of C-shape canal can be classified into biological and technical factors. Failures related to microorganisms can be caused by anatomical difficulties such as isthmus, apical ramification, and other morphological irregularities [16]. The complexity of C-shaped canals makes them difficult to clean, shape, and obturate effectively [17, 18]. Failures also can be caused by procedural errors such as root perforation, separated instruments, or missed canals. The thin dentinal wall of the buccal or lingual groove may lead to strip perforation, which poses a considerable threat to tooth prognosis .One basic factor for the success of root canal treatment is to have adequate knowledge about the shape of the root canal system before endodontic treatment [15]. Two-dimensional images cannot provide sufficient information about the internal complexities of the root canal system and are not often helpful for management of complex cases; but CBCT can reveal dental anomalies and precise anatomy of the root. Therefore, CBCT is considered as the gold standard in diagnosis and treatment planning [2].

The present study evaluated 1231 CBCT scans retrieved from the archives of a private radiology clinic in Hamedan city. The mean age of patients was 33.87 years, and no significant correlation was found between age and root morphology on CBCT scans. Although it has been stated that age may affect the type of root morphology, Funakoshi et al., [6] and Nejaim et al. [3] found no significant correlation between age and root morphology. Yang et al. [19] showed that the frequency of C-shaped canals in those over 61 years (24.08%) was significantly lower than that in 21–30 year-olds (40.02%). They explained that C-shaped canals usually have a complex anatomy which complicates their root canal treatment; therefore, their incidence of extraction increases with age leading to a reduction in the prevalence of C-shaped canals in older age groups.

In the present study, 60.7% of patients were females, and the prevalence of all types of root morphology (types 1–5) and C-shaped canals (69.2%) was higher in females than males. Khawaja et al., [20] Abarca et al., [21] Kantilieraki et al., [22] Martins et al., [8] Mashyakhy et al., [4] and Yang et al. [19] reported significantly higher frequency of C-shaped canals in mandibular second molars of females than males. However, in studies by Aricioğlu et al., [2] and Sarraf et al., [23] a greater number of C-shaped canals was found in males. Both of the above-mentioned studies had a smaller sample size than the present study. The hypothesis of a morphological genetic adaptation in the Asian populations to fit small-sized teeth into smaller jaws is also supported by several systematic studies reporting morphological differences between people of different gender in the same species and helped to make clear to the high prevalence of C-shaped canal morphology in females [8]. In addition, in our study, major part of sample size were belonged to female and this may affect higher prevalence of C-shape canal in the female. In the present study, of 1231 mandibular molar teeth, 772 (62.7%) had type 1 morphology, and the double-root pattern (97.3%) was the most common pattern on the axial CBCT sections. Also, 353 teeth (28.6%) had type 2 morphology; in which, the double-root pattern (92.6%) was more commonly seen on the axial CBCT sections. In the study by Funakoshi et al., [6] 21.1% (223/1058) of the teeth showed type 1 morphology and 43.3% (458/1058) had type 2 morphology, which was consistent with the current findings, and most of them (85%) had a double-root pattern on the axial section. In the study by Khawaja et al., 78.3% of the mandibular second molar teeth had two separate roots (types 1 and 2), and showed the pattern of two separate roots (double-root) on the axial views [20]. In a study by Gomez et al., [24] 85.5% of mandibular second molar teeth had two separate roots and they mostly showed two separate roots on the axial CBCT views.

In the present study, of 1231 mandibular second molar teeth, 48 (3.9%) had type 3 morphology; of which, 47.9% had a C-shaped pattern and 43.8% had a single-root pattern. Also, 11 (0.9%) teeth had type 4 morphology; of which, 45.5% had a single-root pattern. According to Nejaim et al., mandibular second molar teeth with fused roots had 17.2 times higher likelihood of having a C-shaped canal than those with separate roots [3]. In a study by Funakoshi et al., 9.7% (103/1058) had type 3 morphology and 23.8% had type 4 morphology; of which 85% had a C-shaped pattern, especially on sections close to the apex [6]. Difference in the prevalence rates of type 4 morphology, and more common frequency of C-shaped pattern in type 4 morphology in their study compared with the present study may be attributed to different ethnicities of the study populations. The Iranian population was evaluated in the present study while Funakoshi et al. [6] assessed a Japanese population. In the study by Kantilieraki et al., [22] 12.2% of the mandibular second molar teeth had one root with two canals, and 64% had a C-shaped canal on the axial views. Difference in prevalence rates of C-shaped canals in this type of root morphology is probably due to smaller number of samples (524) in their study compared with the present study, and the use of NewTom VGi Evo CBCT scanner with the exposure settings of 110 kVp, 3 mA, and 4.3 s time, which was different from the exposure parameters adopted in the present study.

In the current study, of 1231 mandibular second molar teeth, 47 (3.8%) had type 5 morphology; of which, 76.6% had a single-root pattern. In the study by Funakoshi et al., 2.1% (22/1058) were type 5, and most of them (50%) showed the single-root pattern [6]. Small difference in the prevalence of single-root pattern in their study and the present study is probably due to different ethnicity of their study population (Japanese) and also the difference in the type of CBCT scanner (Alphard Vega scanner Asahi Roentgen Ind. Co. Ltd, Kyoto, Japan), exposure settings of 80 kVp, 8 mA and 17 s time, and use of AquariusNET image processing software (TeraRecon Inc., Foster City, CA) in their study compared with the present study. In the study by Khawaja et al., [20] 2.4% of mandibular second molars had one single root with one canal (type 5 and single-root pattern) on the CBCT axial views. In the study by Nejaim et al., [3] single-root and single-canal teeth mostly (56.8%) had a single-root pattern, and 43.2% of the cases had C-shaped canals.

In the present study, of 772 mandibular second molar teeth with type 1 morphology, 20 (2.6%) teeth had a C-shaped pattern, and in all three cervical, middle and apical sections, they mostly had a C3 pattern (85%, 90% and 90%, respectively). Among 353 mandibular second molar teeth with type 2 morphology, 22 teeth (6.2%) showed a C-shape pattern; among which, C3 pattern was more common (72.7%, 81.8%, and 81.8%, respectively). Of 48 mandibular second molar teeth with type 3 morphology, 23 (47.9%) teeth had a C-shaped pattern. The C1 pattern was more common in the cervical and middle thirds (47.8% and 47.8%, respectively) and the C3 pattern (39.1%) was more frequent in the apical third. Among 11 mandibular second molar teeth with type 4 morphology, 3 (27.3%) had a C-shaped pattern, and all of them only showed C3 pattern in all three sections. Among 47 mandibular second molar teeth with type 5 morphology, 10 teeth (21.3%) had a C-shaped pattern; the C1 and C4 patterns (40% and 40%) were more commonly seen in the cervical third, the C3 pattern (50%) was more frequently seen in the middle third, and the C4 pattern (40%) was more common in the apical third. In the studies by Aricioğlu et al., [2] and Mashyakhy et al., [4] the majority of mandibular second molar teeth with a C-shaped canal had a C3 pattern. In the study by Khawaja et al., mandibular second molar teeth with C-shaped canals mostly had a C1 pattern in the cervical third (41.75%) and they often had C3 pattern in the middle and apical thirds (51.7% and 65.9%, respectively). Also, in 5.5% of the cases with C-shaped canals, the canal pattern did not change along the root, but in 94.5% of them, this pattern changed from the coronal towards the apical, and C1-C2-C3 (18%) was the most common [20]. However, in the present study, the C3 pattern was more commonly seen in the cervical, middle and apical thirds (57.6%, 65.3% and 65.3%, respectively) and in most cases (79.4%) this pattern remained unchanged along the root and no change occurred in C1-C2-C3 shape. This discrepancy may be due to the smaller number of examined samples (508) and different ethnicity of the study populations (Emirati population) as well as the difference in exposure settings.

In the present study, of 1231 examined mandibular second molar teeth, 88.2% had a double-root pattern, 6.3% had a C-shaped pattern, and 5.4% had a single-root pattern. In the study by Sarraf et al., [23] which was conducted on an Iranian population 5.64% of mandibular second molars had a C-shaped pattern, which was consistent with the present findings. The prevalence of C-shaped canals in mandibular second molar teeth was 7.1% in the study by Abarca et al., [16] 5.3% in the study by Kantilieraki et al., [22], 7.9% in the study by Mashyakhy et al., [4] 8.5% in the study by Nejaim et al., [3] and 5.53% in the study by Živanović et al. [25]. The prevalence of C-shaped canals in mandibular second molar teeth was 31.2% in the study by Funakoshi et al., [6], 17.9% in the study by Khawaja et al., [20] and 36.8% in the study by Yang et al. [19]. Different ethnicity of the study populations (Japanese, Emirati, and Korean, respectively) and the difference in CBCT exposure settings are probably responsible for the variations in the results. In the study by Yang et al., [19] 43.9% of the examined mandibular second molar teeth had a C-shaped canal. Different ethnicity of the examined population (South Korean population), smaller sample size, and use of RayScan Alpha Plus CBCT scanner (RAYSCAN Alpha plus; Ray Co., Hwaseong, Korea) with 90 kVp, 12 mA and 14 s time exposure settings in their study are probably the reasons for the difference in the results of the two studies. In future study we recommend systematic review and meta-analysis research about prevalence of C-shape canal in Iranian population and compare of this result with other population.

## Conclusion

According to the present results, type 3 morphology of mandibular second molar teeth on panoramic-like views may indicate high probability of a C-shaped canal. Therefore, in such cases, dental clinicians should preferably request a CBCT for further assessment. If other tooth morphologies are detected on the panoramic-like image (types 1, 2, 4, 5), due to lower prevalence of C-shaped canals in these morphologies, there would be no need to expose patient to additional radiation by requesting a CBCT, and its use should be limited to complex cases. Most of the C-shaped canals seen in the present study had a C3 pattern. Incidence of C-shaped canals (69.2%) was higher in females than males.

## Data Availability

Under reasonable requirements, the data and material of this study can be obtained from the corresponding author.

## Abbreviations

CBCT: Cone-beam computed tomography
